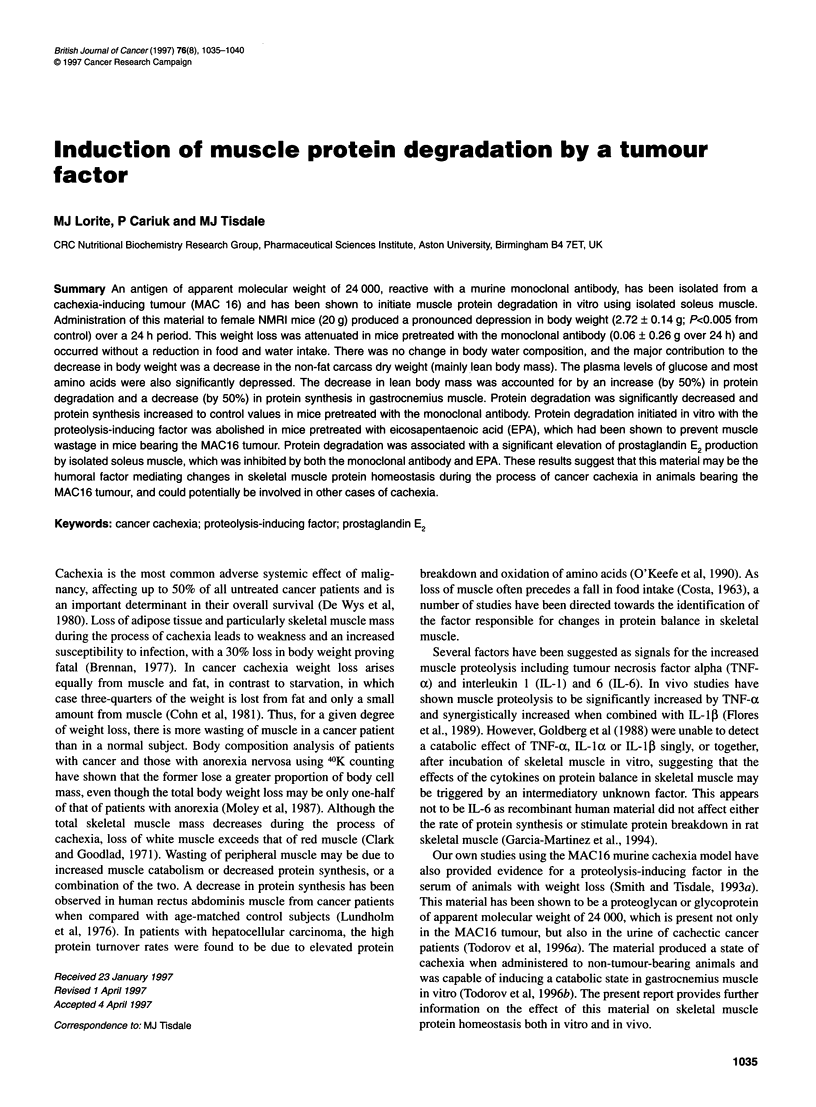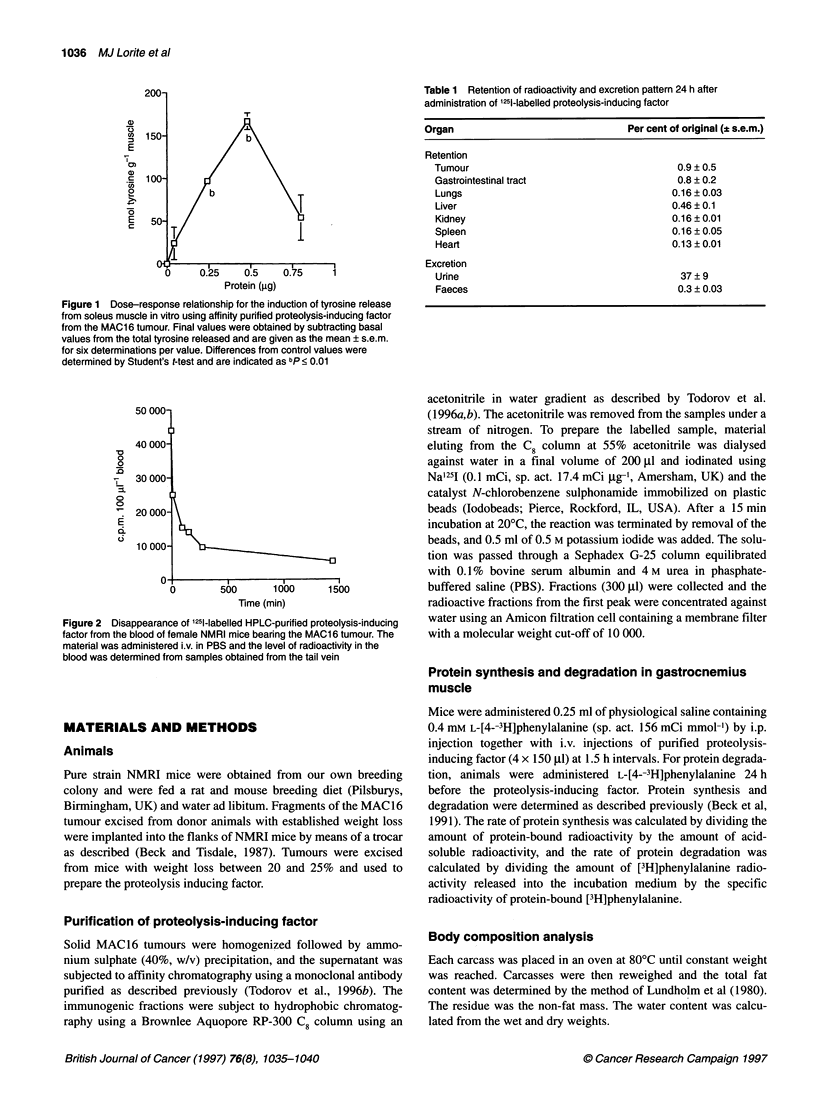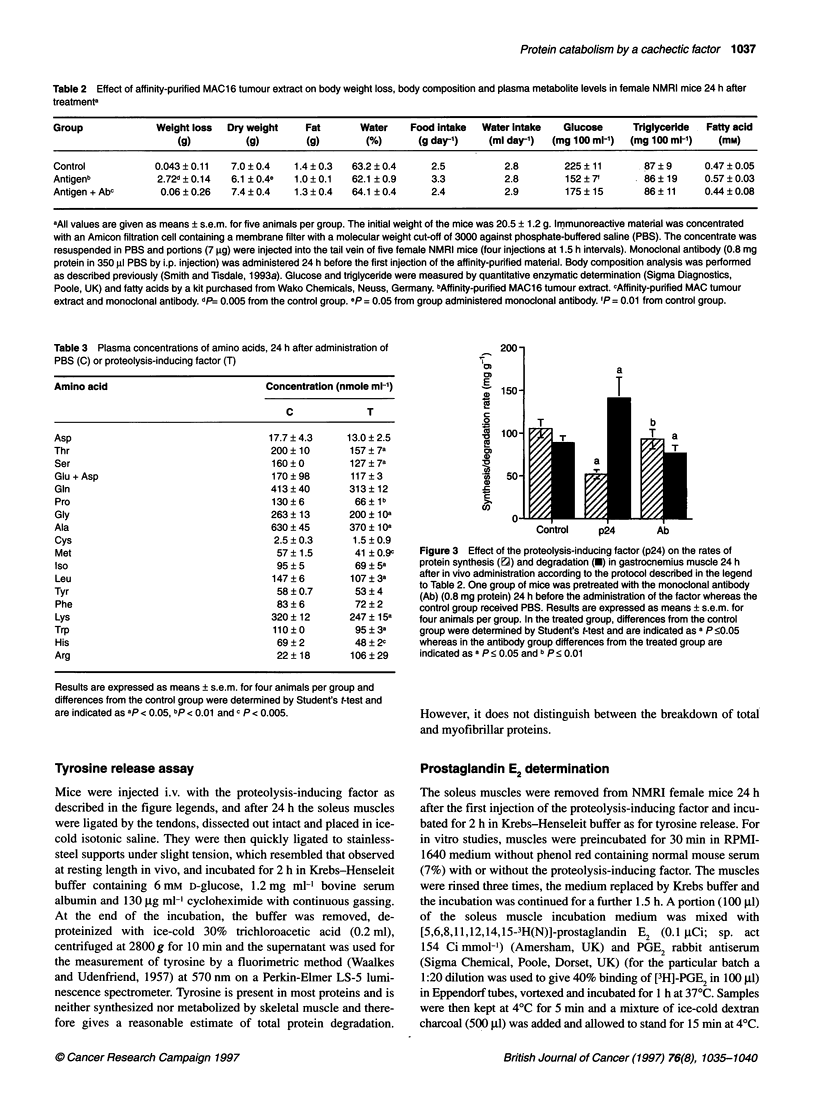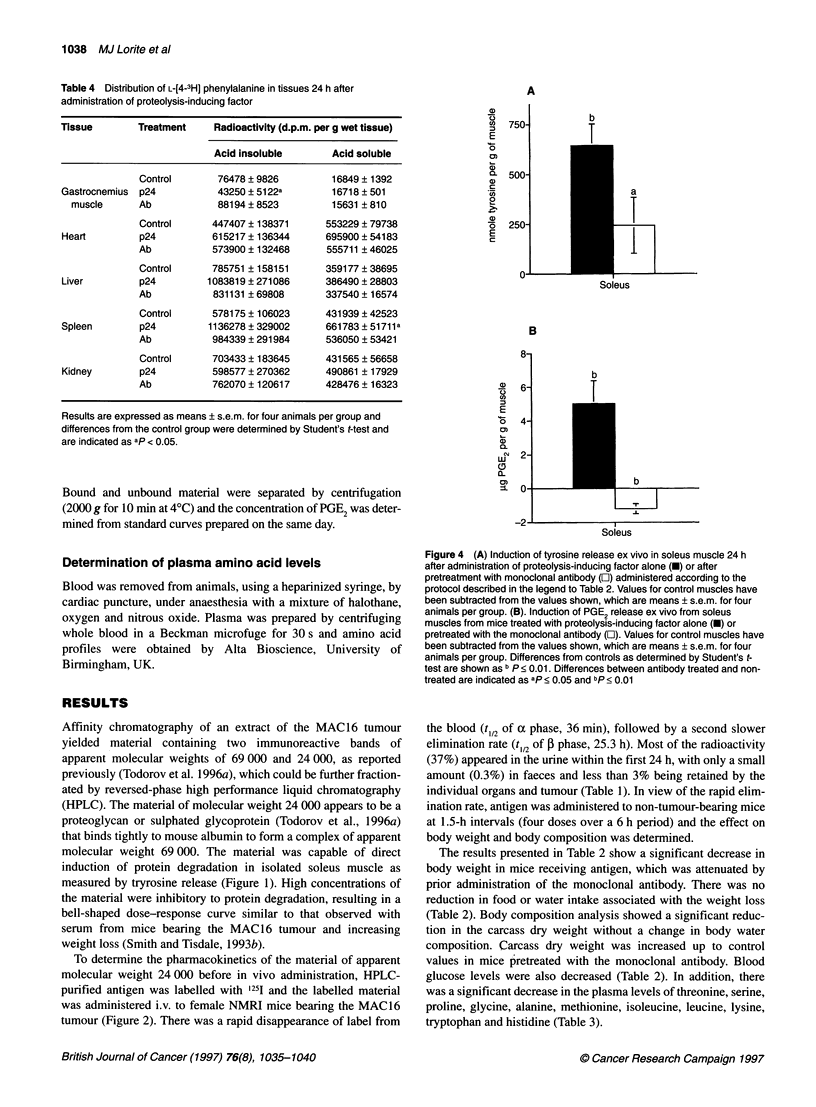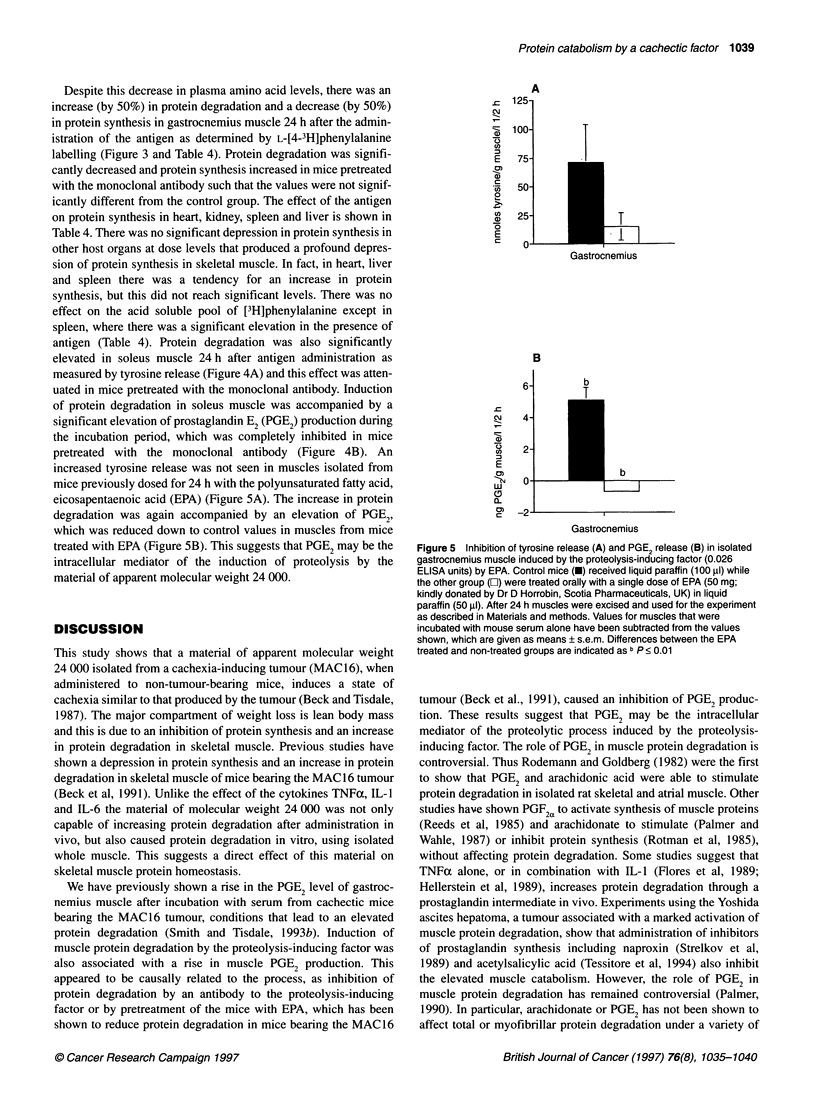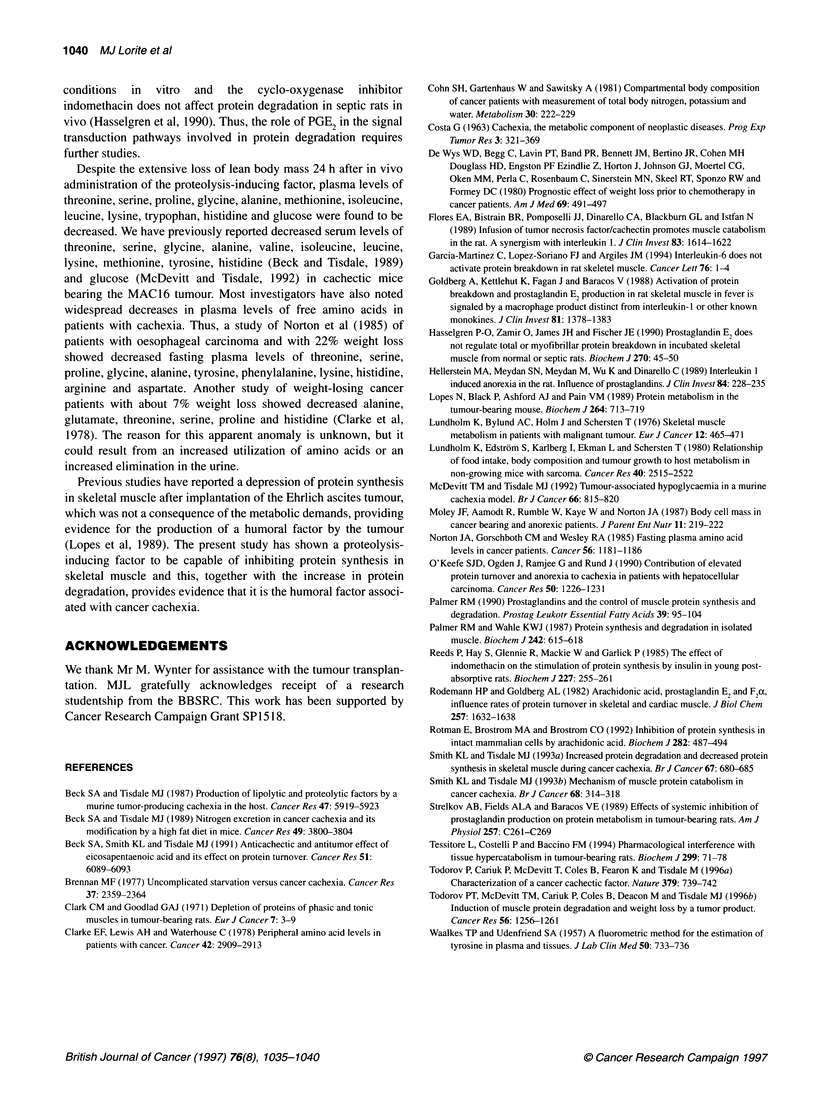# Induction of muscle protein degradation by a tumour factor.

**DOI:** 10.1038/bjc.1997.504

**Published:** 1997

**Authors:** M. J. Lorite, P. Cariuk, M. J. Tisdale

**Affiliations:** CRC Nutritional Biochemistry Research Group, Pharmaceutical Sciences Institute, Aston University, Birmingham, UK.

## Abstract

An antigen of apparent molecular weight of 24,000, reactive with a murine monoclonal antibody, has been isolated from a cachexia-inducing tumour (MAC 16) and has been shown to initiate muscle protein degradation in vitro using isolated soleus muscle. Administration of this material to female NMRI mice (20 g) produced a pronounced depression in body weight (2.72 +/- 0.14 g; P<0.005 from control) over a 24 h period. This weight loss was attenuated in mice pretreated with the monoclonal antibody (0.06 +/- 0.26 g over 24 h) and occurred without a reduction in food and water intake. There was no change in body water composition, and the major contribution to the decrease in body weight was a decrease in the non-fat carcass dry weight (mainly lean body mass). The plasma levels of glucose and most amino acids were also significantly depressed. The decrease in lean body mass was accounted for by an increase (by 50%) in protein degradation and a decrease (by 50%) in protein synthesis in gastrocnemius muscle. Protein degradation was significantly decreased and protein synthesis increased to control values in mice pretreated with the monoclonal antibody. Protein degradation initiated in vitro with the proteolysis-inducing factor was abolished in mice pretreated with eicosapentaenoic acid (EPA), which had been shown to prevent muscle wastage in mice bearing the MAC16 tumour. Protein degradation was associated with a significant elevation of prostaglandin E2 production by isolated soleus muscle, which was inhibited by both the monoclonal antibody and EPA. These results suggest that this material may be the humoral factor mediating changes in skeletal muscle protein homeostasis during the process of cancer cachexia in animals bearing the MAC16 tumour, and could potentially be involved in other cases of cachexia.


British Journal of Cancer (1997) **76**(8), 1035–1040
© 1997 Cancer Research Campaign

### Induction of muscle protein degradation by a tumour factor

**MJ Lorite, P Cariuk and MJ Tisdale**

CRC Nutritional Biochemistry Research Group, Pharmaceutical Sciences Institute, Aston University, Birmingham B4 7ET, UK

**Summary** An antigen of apparent molecular weight of 24 000, reactive with a murine monoclonal antibody, has been isolated from a cachexia-inducing tumour (MAC 16) and has been shown to initiate muscle protein degradation in vitro using isolated soleus muscle. Administration of this material to female NMRI mice (20 g) produced a pronounced depression in body weight (2.72 ± 0.14 g; $P$<0.005 from control) over a 24 h period. This weight loss was attenuated in mice pretreated with the monoclonal antibody (0.06 ± 0.26 g over 24 h) and occurred without a reduction in food and water intake. There was no change in body water composition, and the major contribution to the decrease in body weight was a decrease in the non-fat carcass dry weight (mainly lean body mass). The plasma levels of glucose and most amino acids were also significantly depressed. The decrease in lean body mass was accounted for by an increase (by 50%) in protein degradation and a decrease (by 50%) in protein synthesis in gastrocnemius muscle. Protein degradation was significantly decreased and protein synthesis increased to control values in mice pretreated with the monoclonal antibody. Protein degradation initiated in vitro with the proteolysis-inducing factor was abolished in mice pretreated with eicosapentaenoic acid (EPA), which had been shown to prevent muscle wastage in mice bearing the MAC16 tumour. Protein degradation was associated with a significant elevation of prostaglandin $E_2$ production by isolated soleus muscle, which was inhibited by both the monoclonal antibody and EPA. These results suggest that this material may be the humoral factor mediating changes in skeletal muscle protein homeostasis during the process of cancer cachexia in animals bearing the MAC16 tumour, and could potentially be involved in other cases of cachexia.

**Keywords:** cancer cachexia; proteolysis-inducing factor; prostaglandin $E_2$

Cachexia is the most common adverse systemic effect of malignancy, affecting up to 50% of all untreated cancer patients and is an important determinant in their overall survival (De Wys et al, 1980). Loss of adipose tissue and particularly skeletal muscle mass during the process of cachexia leads to weakness and an increased susceptibility to infection, with a 30% loss in body weight proving fatal (Brennan, 1977). In cancer cachexia weight loss arises equally from muscle and fat, in contrast to starvation, in which case three-quarters of the weight is lost from fat and only a small amount from muscle (Cohn et al, 1981). Thus, for a given degree of weight loss, there is more wasting of muscle in a cancer patient than in a normal subject. Body composition analysis of patients with cancer and those with anorexia nervosa using $^{40}K$ counting have shown that the former lose a greater proportion of body cell mass, even though the total body weight loss may be only one-half of that of patients with anorexia (Moley et al, 1987). Although the total skeletal muscle mass decreases during the process of cachexia, loss of white muscle exceeds that of red muscle (Clark and Goodlad, 1971). Wasting of peripheral muscle may be due to increased muscle catabolism or decreased protein synthesis, or a combination of the two. A decrease in protein synthesis has been observed in human rectus abdominis muscle from cancer patients when compared with age-matched control subjects (Lundholm et al, 1976). In patients with hepatocellular carcinoma, the high protein turnover rates were found to be due to elevated protein breakdown and oxidation of amino acids (O'Keefe et al, 1990). As loss of muscle often precedes a fall in food intake (Costa, 1963), a number of studies have been directed towards the identification of the factor responsible for changes in protein balance in skeletal muscle.

*Received 23 January 1997*
*Revised 1 April 1997*
*Accepted 4 April 1997*

*Correspondence to:* MJ Tisdale

Several factors have been suggested as signals for the increased muscle proteolysis including tumour necrosis factor alpha (TNF-α) and interleukin 1 (IL-1) and 6 (IL-6). In vivo studies have shown muscle proteolysis to be significantly increased by TNF-α and synergistically increased when combined with IL-1β (Flores et al., 1989). However, Goldberg et al (1988) were unable to detect a catabolic effect of TNF-α, IL-1α or IL-1β singly, or together, after incubation of skeletal muscle in vitro, suggesting that the effects of the cytokines on protein balance in skeletal muscle may be triggered by an intermediatory unknown factor. This appears not to be IL-6 as recombinant human material did not affect either the rate of protein synthesis or stimulate protein breakdown in rat skeletal muscle (Garcia-Martinez et al., 1994).

Our own studies using the MAC16 murine cachexia model have also provided evidence for a proteolysis-inducing factor in the serum of animals with weight loss (Smith and Tisdale, 1993*a*). This material has been shown to be a proteoglycan or glycoprotein of apparent molecular weight of 24 000, which is present not only in the MAC16 tumour, but also in the urine of cachectic cancer patients (Todorov et al, 1996*a*). The material produced a state of cachexia when administered to non-tumour-bearing animals and was capable of inducing a catabolic state in gastrocnemius muscle in vitro (Todorov et al, 1996*b*). The present report provides further information on the effect of this material on skeletal muscle protein homeostasis both in vitro and in vivo.

**Figure 1** Dose–response relationship for the induction of tyrosine release from soleus muscle in vitro using affinity purified proteolysis-inducing factor from the MAC16 tumour. Final values were obtained by subtracting basal values from the total tyrosine released and are given as the mean ± s.e.m. for six determinations per value. Differences from control values were determined by Student's *t*-test and are indicated as $^{b}P \leq 0.01$

**Table 1** Retention of radioactivity and excretion pattern 24 h after administration of $^{125}$I-labelled proteolysis-inducing factor

| Organ | Per cent of original (± s.e.m.) |
|---|---|
| Retention | |
| Tumour | 0.9 ± 0.5 |
| Gastrointestinal tract | 0.8 ± 0.2 |
| Lungs | 0.16 ± 0.03 |
| Liver | 0.46 ± 0.1 |
| Kidney | 0.16 ± 0.01 |
| Spleen | 0.16 ± 0.05 |
| Heart | 0.13 ± 0.01 |
| Excretion | |
| Urine | 37 ± 9 |
| Faeces | 0.3 ± 0.03 |

**Figure 2** Disappearance of $^{125}$I-labelled HPLC-purified proteolysis-inducing factor from the blood of female NMRI mice bearing the MAC16 tumour. The material was administered i.v. in PBS and the level of radioactivity in the blood was determined from samples obtained from the tail vein

### MATERIALS AND METHODS

### Animals

Pure strain NMRI mice were obtained from our own breeding colony and were fed a rat and mouse breeding diet (Pilsburys, Birmingham, UK) and water ad libitum. Fragments of the MAC16 tumour excised from donor animals with established weight loss were implanted into the flanks of NMRI mice by means of a trocar as described (Beck and Tisdale, 1987). Tumours were excised from mice with weight loss between 20 and 25% and used to prepare the proteolysis inducing factor.

### Purification of proteolysis-inducing factor

Solid MAC16 tumours were homogenized followed by ammonium sulphate (40%, w/v) precipitation, and the supernatant was subjected to affinity chromatography using a monoclonal antibody purified as described previously (Todorov et al., 1996*b*). The immunogenic fractions were subject to hydrophobic chromatography using a Brownlee Aquopore RP-300 $C_8$ column using an acetonitrile in water gradient as described by Todorov et al. (1996*a,b*). The acetonitrile was removed from the samples under a stream of nitrogen. To prepare the labelled sample, material eluting from the $C_8$ column at 55% acetonitrile was dialysed against water in a final volume of 200 μl and iodinated using Na$^{125}$I (0.1 mCi, sp. act. 17.4 mCi μg$^{-1}$, Amersham, UK) and the catalyst *N*-chlorobenzene sulphonamide immobilized on plastic beads (Iodobeads; Pierce, Rockford, IL, USA). After a 15 min incubation at 20°C, the reaction was terminated by removal of the beads, and 0.5 ml of 0.5 M potassium iodide was added. The solution was passed through a Sephadex G-25 column equilibrated with 0.1% bovine serum albumin and 4 M urea in phosphate-buffered saline (PBS). Fractions (300 μl) were collected and the radioactive fractions from the first peak were concentrated against water using an Amicon filtration cell containing a membrane filter with a molecular weight cut-off of 10 000.

### Protein synthesis and degradation in gastrocnemius muscle

Mice were administered 0.25 ml of physiological saline containing 0.4 mM L-[4-$^{3}$H]phenylalanine (sp. act. 156 mCi mmol$^{-1}$) by i.p. injection together with i.v. injections of purified proteolysis-inducing factor (4 × 150 μl) at 1.5 h intervals. For protein degradation, animals were administered L-[4-$^{3}$H]phenylalanine 24 h before the proteolysis-inducing factor. Protein synthesis and degradation were determined as described previously (Beck et al, 1991). The rate of protein synthesis was calculated by dividing the amount of protein-bound radioactivity by the amount of acid-soluble radioactivity, and the rate of protein degradation was calculated by dividing the amount of [$^{3}$H]phenylalanine radioactivity released into the incubation medium by the specific radioactivity of protein-bound [$^{3}$H]phenylalanine.

### Body composition analysis

Each carcass was placed in an oven at 80°C until constant weight was reached. Carcasses were then reweighed and the total fat content was determined by the method of Lundholm et al (1980). The residue was the non-fat mass. The water content was calculated from the wet and dry weights.

© Cancer Research Campaign 1997

**Table 2** Effect of affinity-purified MAC16 tumour extract on body weight loss, body composition and plasma metabolite levels in female NMRI mice 24 h after treatment[a]

| Group | Weight loss (g) | Dry weight (g) | Fat (g) | Water (%) | Food intake (g day$^{-1}$) | Water intake (ml day$^{-1}$) | Glucose (mg 100 ml$^{-1}$) | Triglyceride (mg 100 ml$^{-1}$) | Fatty acid (mM) |
|---|---|---|---|---|---|---|---|---|---|
| Control | 0.043 ± 0.11 | 7.0 ± 0.4 | 1.4 ± 0.3 | 63.2 ± 0.4 | 2.5 | 2.8 | 225 ± 11 | 87 ± 9 | 0.47 ± 0.05 |
| Antigen[b] | 2.72[d] ± 0.14 | 6.1 ± 0.4[e] | 1.0 ± 0.1 | 62.1 ± 0.9 | 3.3 | 2.8 | 152 ± 7[f] | 86 ± 19 | 0.57 ± 0.03 |
| Antigen + Ab[c] | 0.06 ± 0.26 | 7.4 ± 0.4 | 1.3 ± 0.4 | 64.1 ± 0.4 | 2.4 | 2.9 | 175 ± 15 | 86 ± 11 | 0.44 ± 0.08 |

[a]All values are given as means ± s.e.m. for five animals per group. The initial weight of the mice was 20.5 ± 1.2 g. Immunoreactive material was concentrated with an Amicon filtration cell containing a membrane filter with a molecular weight cut-off of 3000 against phosphate-buffered saline (PBS). The concentrate was resuspended in PBS and portions (7 μg) were injected into the tail vein of five female NMRI mice (four injections at 1.5 h intervals). Monoclonal antibody (0.8 mg protein in 350 μl PBS by i.p. injection) was administered 24 h before the first injection of the affinity-purified material. Body composition analysis was performed as described previously (Smith and Tisdale, 1993*a*). Glucose and triglyceride were measured by quantitative enzymatic determination (Sigma Diagnostics, Poole, UK) and fatty acids by a kit purchased from Wako Chemicals, Neuss, Germany. [b]Affinity-purified MAC16 tumour extract. [c]Affinity-purified MAC tumour extract and monoclonal antibody. [d]$P$= 0.005 from the control group. [e]$P$ = 0.05 from group administered monoclonal antibody. [f]$P$ = 0.01 from control group.

**Table 3** Plasma concentrations of amino acids, 24 h after administration of PBS (C) or proteolysis-inducing factor (T)

| Amino acid | Concentration (nmole ml$^{-1}$) | |
|---|---|---|
| | C | T |
| Asp | 17.7 ± 4.3 | 13.0 ± 2.5 |
| Thr | 200 ± 10 | 157 ± 7[a] |
| Ser | 160 ± 0 | 127 ± 7[a] |
| Glu + Asp | 170 ± 98 | 117 ± 3 |
| Gln | 413 ± 40 | 313 ± 12 |
| Pro | 130 ± 6 | 66 ± 1[b] |
| Gly | 263 ± 13 | 200 ± 10[a] |
| Ala | 630 ± 45 | 370 ± 10[a] |
| Cys | 2.5 ± 0.3 | 1.5 ± 0.9 |
| Met | 57 ± 1.5 | 41 ± 0.9[c] |
| Iso | 95 ± 5 | 69 ± 5[a] |
| Leu | 147 ± 6 | 107 ± 3[a] |
| Tyr | 58 ± 0.7 | 53 ± 4 |
| Phe | 83 ± 6 | 72 ± 2 |
| Lys | 320 ± 12 | 247 ± 15[a] |
| Trp | 110 ± 0 | 95 ± 3[a] |
| His | 69 ± 2 | 48 ± 2[c] |
| Arg | 22 ± 18 | 106 ± 29 |

Results are expressed as means ± s.e.m. for four animals per group and differences from the control group were determined by Student's *t*-test and are indicated as [a]$P$ < 0.05, [b]$P$ < 0.01 and [c] $P$ < 0.005.

**Figure 3** Effect of the proteolysis-inducing factor (p24) on the rates of protein synthesis (▨) and degradation (■) in gastrocnemius muscle 24 h after in vivo administration according to the protocol described in the legend to Table 2. One group of mice was pretreated with the monoclonal antibody (Ab) (0.8 mg protein) 24 h before the administration of the factor whereas the control group received PBS. Results are expressed as means ± s.e.m. for four animals per group. In the treated group, differences from the control group were determined by Student's *t*-test and are indicated as [a] $P \leq 0.05$ whereas in the antibody group differences from the treated group are indicated as [a] $P \leq 0.05$ and [b] $P \leq 0.01$

### Tyrosine release assay

Mice were injected i.v. with the proteolysis-inducing factor as described in the figure legends, and after 24 h the soleus muscles were ligated by the tendons, dissected out intact and placed in ice-cold isotonic saline. They were then quickly ligated to stainless-steel supports under slight tension, which resembled that observed at resting length in vivo, and incubated for 2 h in Krebs–Henseleit buffer containing 6 mM D-glucose, 1.2 mg ml$^{-1}$ bovine serum albumin and 130 μg ml$^{-1}$ cycloheximide with continuous gassing. At the end of the incubation, the buffer was removed, deproteinized with ice-cold 30% trichloroacetic acid (0.2 ml), centrifuged at 2800 ***g*** for 10 min and the supernatant was used for the measurement of tyrosine by a fluorimetric method (Waalkes and Udenfriend, 1957) at 570 nm on a Perkin-Elmer LS-5 luminescence spectrometer. Tyrosine is present in most proteins and is neither synthesized nor metabolized by skeletal muscle and therefore gives a reasonable estimate of total protein degradation. However, it does not distinguish between the breakdown of total and myofibrillar proteins.

### Prostaglandin $E_2$ determination

The soleus muscles were removed from NMRI female mice 24 h after the first injection of the proteolysis-inducing factor and incubated for 2 h in Krebs–Henseleit buffer as for tyrosine release. For in vitro studies, muscles were preincubated for 30 min in RPMI-1640 medium without phenol red containing normal mouse serum (7%) with or without the proteolysis-inducing factor. The muscles were rinsed three times, the medium replaced by Krebs buffer and the incubation was continued for a further 1.5 h. A portion (100 μl) of the soleus muscle incubation medium was mixed with [5,6,8,11,12,14,15-$^3$H(N)]-prostaglandin $E_2$ (0.1 μCi; sp. act 154 Ci mmol$^{-1}$) (Amersham, UK) and $PGE_2$ rabbit antiserum (Sigma Chemical, Poole, Dorset, UK) (for the particular batch a 1:20 dilution was used to give 40% binding of [$^3$H]-$PGE_2$ in 100 μl) in Eppendorf tubes, vortexed and incubated for 1 h at 37°C. Samples were then kept at 4°C for 5 min and a mixture of ice-cold dextran charcoal (500 μl) was added and allowed to stand for 15 min at 4°C.

© Cancer Research Campaign 1997

**Table 4** Distribution of L-[4-$^3$H] phenylalanine in tissues 24 h after administration of proteolysis-inducing factor

| Tissue | Treatment | Radioactivity (d.p.m. per g wet tissue) | |
|---|---|---|---|
| | | Acid insoluble | Acid soluble |
| | Control | 76478 ± 9826 | 16849 ± 1392 |
| Gastrocnemius | p24 | 43250 ± 5122[a] | 16718 ± 501 |
| muscle | Ab | 88194 ± 8523 | 15631 ± 810 |
| | Control | 447407 ± 138371 | 553229 ± 79738 |
| Heart | p24 | 615217 ± 136344 | 695900 ± 54183 |
| | Ab | 573900 ± 132468 | 555711 ± 46025 |
| | Control | 785751 ± 158151 | 359177 ± 38695 |
| Liver | p24 | 1083819 ± 271086 | 386490 ± 28803 |
| | Ab | 831131 ± 69808 | 337540 ± 16574 |
| | Control | 578175 ± 106023 | 431939 ± 42523 |
| Spleen | p24 | 1136278 ± 329002 | 661783 ± 51711[a] |
| | Ab | 984339 ± 291984 | 536050 ± 53421 |
| | Control | 703433 ± 183645 | 431565 ± 56658 |
| Kidney | p24 | 598577 ± 270362 | 490861 ± 17929 |
| | Ab | 762070 ± 120617 | 428476 ± 16323 |

Results are expressed as means ± s.e.m. for four animals per group and differences from the control group were determined by Student's *t*-test and are indicated as [a]$P < 0.05$.

Bound and unbound material were separated by centrifugation (2000 *g* for 10 min at 4°C) and the concentration of $PGE_2$ was determined from standard curves prepared on the same day.

### Determination of plasma amino acid levels

Blood was removed from animals, using a heparinized syringe, by cardiac puncture, under anaesthesia with a mixture of halothane, oxygen and nitrous oxide. Plasma was prepared by centrifuging whole blood in a Beckman microfuge for 30 s and amino acid profiles were obtained by Alta Bioscience, University of Birmingham, UK.

**Figure 4** (**A**) Induction of tyrosine release ex vivo in soleus muscle 24 h after administration of proteolysis-inducing factor alone (■) or after pretreatment with monoclonal antibody (□) administered according to the protocol described in the legend to Table 2. Values for control muscles have been subtracted from the values shown, which are means ± s.e.m. for four animals per group. (**B**). Induction of $PGE_2$ release ex vivo from soleus muscles from mice treated with proteolysis-inducing factor alone (■) or pretreated with the monoclonal antibody (□). Values for control muscles have been subtracted from the values shown, which are means ± s.e.m. for four animals per group. Differences from controls as determined by Student's *t*-test are shown as [b] $P \leq 0.01$. Differences between antibody treated and non-treated are indicated as [a]$P \leq 0.05$ and [b]$P \leq 0.01$

### RESULTS

Affinity chromatography of an extract of the MAC16 tumour yielded material containing two immunoreactive bands of apparent molecular weights of 69 000 and 24 000, as reported previously (Todorov et al. 1996*a*), which could be further fractionated by reversed-phase high performance liquid chromatography (HPLC). The material of molecular weight 24 000 appears to be a proteoglycan or sulphated glycoprotein (Todorov et al., 1996*a*) that binds tightly to mouse albumin to form a complex of apparent molecular weight 69 000. The material was capable of direct induction of protein degradation in isolated soleus muscle as measured by tyrosine release (Figure 1). High concentrations of the material were inhibitory to protein degradation, resulting in a bell-shaped dose–response curve similar to that observed with serum from mice bearing the MAC16 tumour and increasing weight loss (Smith and Tisdale, 1993*b*).

To determine the pharmacokinetics of the material of apparent molecular weight 24 000 before in vivo administration, HPLC-purified antigen was labelled with $^{125}I$ and the labelled material was administered i.v. to female NMRI mice bearing the MAC16 tumour (Figure 2). There was a rapid disappearance of label from the blood ($t_{1/2}$ of α phase, 36 min), followed by a second slower elimination rate ($t_{1/2}$ of β phase, 25.3 h). Most of the radioactivity (37%) appeared in the urine within the first 24 h, with only a small amount (0.3%) in faeces and less than 3% being retained by the individual organs and tumour (Table 1). In view of the rapid elimination rate, antigen was administered to non-tumour-bearing mice at 1.5-h intervals (four doses over a 6 h period) and the effect on body weight and body composition was determined.

The results presented in Table 2 show a significant decrease in body weight in mice receiving antigen, which was attenuated by prior administration of the monoclonal antibody. There was no reduction in food or water intake associated with the weight loss (Table 2). Body composition analysis showed a significant reduction in the carcass dry weight without a change in body water composition. Carcass dry weight was increased up to control values in mice pretreated with the monoclonal antibody. Blood glucose levels were also decreased (Table 2). In addition, there was a significant decrease in the plasma levels of threonine, serine, proline, glycine, alanine, methionine, isoleucine, leucine, lysine, tryptophan and histidine (Table 3).

© Cancer Research Campaign 1997

Despite this decrease in plasma amino acid levels, there was an increase (by 50%) in protein degradation and a decrease (by 50%) in protein synthesis in gastrocnemius muscle 24 h after the administration of the antigen as determined by L-[4-$^3$H]phenylalanine labelling (Figure 3 and Table 4). Protein degradation was significantly decreased and protein synthesis increased in mice pretreated with the monoclonal antibody such that the values were not significantly different from the control group. The effect of the antigen on protein synthesis in heart, kidney, spleen and liver is shown in Table 4. There was no significant depression in protein synthesis in other host organs at dose levels that produced a profound depression of protein synthesis in skeletal muscle. In fact, in heart, liver and spleen there was a tendency for an increase in protein synthesis, but this did not reach significant levels. There was no effect on the acid soluble pool of [$^3$H]phenylalanine except in spleen, where there was a significant elevation in the presence of antigen (Table 4). Protein degradation was also significantly elevated in soleus muscle 24 h after antigen administration as measured by tyrosine release (Figure 4A) and this effect was attenuated in mice pretreated with the monoclonal antibody. Induction of protein degradation in soleus muscle was accompanied by a significant elevation of prostaglandin $E_2$ ($PGE_2$) production during the incubation period, which was completely inhibited in mice pretreated with the monoclonal antibody (Figure 4B). An increased tyrosine release was not seen in muscles isolated from mice previously dosed for 24 h with the polyunsaturated fatty acid, eicosapentaenoic acid (EPA) (Figure 5A). The increase in protein degradation was again accompanied by an elevation of $PGE_2$, which was reduced down to control values in muscles from mice treated with EPA (Figure 5B). This suggests that $PGE_2$ may be the intracellular mediator of the induction of proteolysis by the material of apparent molecular weight 24 000.

**Figure 5** Inhibition of tyrosine release (**A**) and $PGE_2$ release (**B**) in isolated gastrocnemius muscle induced by the proteolysis-inducing factor (0.026 ELISA units) by EPA. Control mice (■) received liquid paraffin (100 µl) while the other group (□) were treated orally with a single dose of EPA (50 mg; kindly donated by Dr D Horrobin, Scotia Pharmaceuticals, UK) in liquid paraffin (50 µl). After 24 h muscles were excised and used for the experiment as described in Materials and methods. Values for muscles that were incubated with mouse serum alone have been subtracted from the values shown, which are given as means ± s.e.m. Differences between the EPA treated and non-treated groups are indicated as $^b$ $P \leq 0.01$

### DISCUSSION

This study shows that a material of apparent molecular weight 24 000 isolated from a cachexia-inducing tumour (MAC16), when administered to non-tumour-bearing mice, induces a state of cachexia similar to that produced by the tumour (Beck and Tisdale, 1987). The major compartment of weight loss is lean body mass and this is due to an inhibition of protein synthesis and an increase in protein degradation in skeletal muscle. Previous studies have shown a depression in protein synthesis and an increase in protein degradation in skeletal muscle of mice bearing the MAC16 tumour (Beck et al, 1991). Unlike the effect of the cytokines TNFα, IL-1 and IL-6 the material of molecular weight 24 000 was not only capable of increasing protein degradation after administration in vivo, but also caused protein degradation in vitro, using isolated whole muscle. This suggests a direct effect of this material on skeletal muscle protein homeostasis.

We have previously shown a rise in the $PGE_2$ level of gastrocnemius muscle after incubation with serum from cachectic mice bearing the MAC16 tumour, conditions that lead to an elevated protein degradation (Smith and Tisdale, 1993*b*). Induction of muscle protein degradation by the proteolysis-inducing factor was also associated with a rise in muscle $PGE_2$ production. This appeared to be causally related to the process, as inhibition of protein degradation by an antibody to the proteolysis-inducing factor or by pretreatment of the mice with EPA, which has been shown to reduce protein degradation in mice bearing the MAC16 tumour (Beck et al., 1991), caused an inhibition of $PGE_2$ production. These results suggest that $PGE_2$ may be the intracellular mediator of the proteolytic process induced by the proteolysis-inducing factor. The role of $PGE_2$ in muscle protein degradation is controversial. Thus Rodemann and Goldberg (1982) were the first to show that $PGE_2$ and arachidonic acid were able to stimulate protein degradation in isolated rat skeletal and atrial muscle. Other studies have shown $PGF_{2\alpha}$ to activate synthesis of muscle proteins (Reeds et al, 1985) and arachidonate to stimulate (Palmer and Wahle, 1987) or inhibit protein synthesis (Rotman et al, 1985), without affecting protein degradation. Some studies suggest that TNFα alone, or in combination with IL-1 (Flores et al, 1989; Hellerstein et al, 1989), increases protein degradation through a prostaglandin intermediate in vivo. Experiments using the Yoshida ascites hepatoma, a tumour associated with a marked activation of muscle protein degradation, show that administration of inhibitors of prostaglandin synthesis including naproxin (Strelkov et al, 1989) and acetylsalicylic acid (Tessitore et al, 1994) also inhibit the elevated muscle catabolism. However, the role of $PGE_2$ in muscle protein degradation has remained controversial (Palmer, 1990). In particular, arachidonate or $PGE_2$ has not been shown to affect total or myofibrillar protein degradation under a variety of

© Cancer Research Campaign 1997

conditions in vitro and the cyclo-oxygenase inhibitor indomethacin does not affect protein degradation in septic rats in vivo (Hasselgren et al, 1990). Thus, the role of $PGE_2$ in the signal transduction pathways involved in protein degradation requires further studies.

Despite the extensive loss of lean body mass 24 h after in vivo administration of the proteolysis-inducing factor, plasma levels of threonine, serine, proline, glycine, alanine, methionine, isoleucine, leucine, lysine, trypophan, histidine and glucose were found to be decreased. We have previously reported decreased serum levels of threonine, serine, glycine, alanine, valine, isoleucine, leucine, lysine, methionine, tyrosine, histidine (Beck and Tisdale, 1989) and glucose (McDevitt and Tisdale, 1992) in cachectic mice bearing the MAC16 tumour. Most investigators have also noted widespread decreases in plasma levels of free amino acids in patients with cachexia. Thus, a study of Norton et al (1985) of patients with oesophageal carcinoma and with 22% weight loss showed decreased fasting plasma levels of threonine, serine, proline, glycine, alanine, tyrosine, phenylalanine, lysine, histidine, arginine and aspartate. Another study of weight-losing cancer patients with about 7% weight loss showed decreased alanine, glutamate, threonine, serine, proline and histidine (Clarke et al, 1978). The reason for this apparent anomaly is unknown, but it could result from an increased utilization of amino acids or an increased elimination in the urine.

Previous studies have reported a depression of protein synthesis in skeletal muscle after implantation of the Ehrlich ascites tumour, which was not a consequence of the metabolic demands, providing evidence for the production of a humoral factor by the tumour (Lopes et al, 1989). The present study has shown a proteolysis-inducing factor to be capable of inhibiting protein synthesis in skeletal muscle and this, together with the increase in protein degradation, provides evidence that it is the humoral factor associated with cancer cachexia.

### ACKNOWLEDGEMENTS

We thank Mr M. Wynter for assistance with the tumour transplantation. MJL gratefully acknowledges receipt of a research studentship from the BBSRC. This work has been supported by Cancer Research Campaign Grant SP1518.

### REFERENCES

Beck SA and Tisdale MJ (1987) Production of lipolytic and proteolytic factors by a murine tumor-producing cachexia in the host. *Cancer Res* **47**: 5919–5923

Beck SA and Tisdale MJ (1989) Nitrogen excretion in cancer cachexia and its modification by a high fat diet in mice. *Cancer Res* **49**: 3800–3804

Beck SA, Smith KL and Tisdale MJ (1991) Anticachectic and antitumor effect of eicosapentaenoic acid and its effect on protein turnover. *Cancer Res* **51**: 6089–6093

Brennan MF (1977) Uncomplicated starvation versus cancer cachexia. *Cancer Res* **37**: 2359–2364

Clark CM and Goodlad GAJ (1971) Depletion of proteins of phasic and tonic muscles in tumour-bearing rats. *Eur J Cancer* **7**: 3–9

Clarke EF, Lewis AH and Waterhouse C (1978) Peripheral amino acid levels in patients with cancer. *Cancer* **42**: 2909–2913

Cohn SH, Gartenhaus W and Sawitsky A (1981) Compartmental body composition of cancer patients with measurement of total body nitrogen, potassium and water. *Metabolism* **30**: 222–229

Costa G (1963) Cachexia, the metabolic component of neoplastic diseases. *Prog Exp Tumor Res* **3**: 321–369

De Wys WD, Begg C, Lavin PT, Band PR, Bennett JM, Bertino JR, Cohen MH Douglass HD, Engston PF Ezindlie Z, Horton J, Johnson GJ, Moertel CG, Oken MM, Perla C, Rosenbaum C, Sinerstein MN, Skeel RT, Sponzo RW and Formey DC (1980) Prognostic effect of weight loss prior to chemotherapy in cancer patients. *Am J Med* **69**: 491–497

Flores EA, Bistrain BR, Pomposelli JJ, Dinarello CA, Blackburn GL and Istfan N (1989) Infusion of tumor necrosis factor/cachectin promotes muscle catabolism in the rat. A synergism with interleukin 1. *J Clin Invest* **83**: 1614–1622

Garcia-Martinez C, Lopez-Soriano FJ and Argiles JM (1994) Interleukin-6 does not activate protein breakdown in rat skeletel muscle. *Cancer Lett* **76**: 1–4

Goldberg A, Kettlehut K, Fagan J and Baracos V (1988) Activation of protein breakdown and prostaglandin $E_2$ production in rat skeletal muscle in fever is signaled by a macrophage product distinct from interleukin-1 or other known monokines. *J Clin Invest* **81**: 1378–1383

Hasselgren P-O, Zamir O, James JH and Fischer JE (1990) Prostaglandin $E_2$ does not regulate total or myofibrillar protein breakdown in incubated skeletal muscle from normal or septic rats. *Biochem J* **270**: 45–50

Hellerstein MA, Meydan SN, Meydan M, Wu K and Dinarello C (1989) Interleukin 1 induced anorexia in the rat. Influence of prostaglandins. *J Clin Invest* **84**: 228–235

Lopes N, Black P, Ashford AJ and Pain VM (1989) Protein metabolism in the tumour-bearing mouse. *Biochem J* **264**: 713–719

Lundholm K, Bylund AC, Holm J and Schersten T (1976) Skeletal muscle metabolism in patients with malignant tumour. *Eur J Cancer* **12**: 465–471

Lundholm K, Edström S, Karlberg I, Ekman L and Schersten T (1980) Relationship of food intake, body composition and tumour growth to host metabolism in non-growing mice with sarcoma. *Cancer Res* **40**: 2515–2522

McDevitt TM and Tisdale MJ (1992) Tumour-associated hypoglycaemia in a murine cachexia model. *Br J Cancer* **66**: 815–820

Moley JF, Aamodt R, Rumble W, Kaye W and Norton JA (1987) Body cell mass in cancer bearing and anorexic patients. *J Parent Ent Nutr* **11**: 219–222

Norton JA, Gorschboth CM and Wesley RA (1985) Fasting plasma amino acid levels in cancer patients. *Cancer* **56**: 1181–1186

O'Keefe SJD, Ogden J, Ramjee G and Rund J (1990) Contribution of elevated protein turnover and anorexia to cachexia in patients with hepatocellular carcinoma. *Cancer Res* **50**: 1226–1231

Palmer RM (1990) Prostaglandins and the control of muscle protein synthesis and degradation. *Prostag Leukotr Essential Fatty Acids* **39**: 95–104

Palmer RM and Wahle KWJ (1987) Protein synthesis and degradation in isolated muscle. *Biochem J* **242**: 615–618

Reeds P, Hay S, Glennie R, Mackie W and Garlick P (1985) The effect of indomethacin on the stimulation of protein synthesis by insulin in young post-absorptive rats. *Biochem J* **227**: 255–261

Rodemann HP and Goldberg AL (1982) Arachidonic acid, prostaglandin $E_2$ and $F_2\alpha$, influence rates of protein turnover in skeletal and cardiac muscle. *J Biol Chem* **257**: 1632–1638

Rotman E, Brostrom MA and Brostrom CO (1992) Inhibition of protein synthesis in intact mammalian cells by arachidonic acid. *Biochem J* **282**: 487–494

Smith KL and Tisdale MJ (1993*a*) Increased protein degradation and decreased protein synthesis in skeletal muscle during cancer cachexia. *Br J Cancer* **67**: 680–685

Smith KL and Tisdale MJ (1993*b*) Mechanism of muscle protein catabolism in cancer cachexia. *Br J Cancer* **68**: 314–318

Strelkov AB, Fields ALA and Baracos VE (1989) Effects of systemic inhibition of prostaglandin production on protein metabolism in tumour-bearing rats. *Am J Physiol* **257**: C261–C269

Tessitore L, Costelli P and Baccino FM (1994) Pharmacological interference with tissue hypercatabolism in tumour-bearing rats. *Biochem J* **299**: 71–78

Todorov P, Cariuk P, McDevitt T, Coles B, Fearon K and Tisdale M (1996*a*) Characterization of a cancer cachectic factor. *Nature* **379**: 739–742

Todorov PT, McDevitt TM, Cariuk P, Coles B, Deacon M and Tisdale MJ (1996*b*) Induction of muscle protein degradation and weight loss by a tumor product. *Cancer Res* **56**: 1256–1261

Waalkes TP and Udenfriend SA (1957) A fluorometric method for the estimation of tyrosine in plasma and tissues. *J Lab Clin Med* **50**: 733–736

 © Cancer Research Campaign 1997